# MYH9 and APOL1 Gene Polymorphisms and the Risk of CKD in Patients with Lupus Nephritis from an Admixture Population

**DOI:** 10.1371/journal.pone.0087716

**Published:** 2014-03-21

**Authors:** Vinícius Sardão Colares, Silvia Maria de Oliveira Titan, Alexandre da Costa Pereira, Patrícia Malafronte, Mari M. Cardena, Sidney Santos, Paulo C. Santos, Cíntia Fridman, Rui Toledo Barros, Viktória Woronik

**Affiliations:** 1 Nephrology Division, Hospital das Clínicas, Sao Paulo University Medical School, Sao Paulo, Sao Paulo, Brazil; 2 Molecular Cardiology Laboratory, Heart Institute (InCor), Sao Paulo University Medical School, Sao Paulo, Sao Paulo, Brazil; 3 Department of Legal Medicine, Ethics and Occupational Health, Medical School, University of São Paulo, Sao Paulo, Sao Paulo, Brazil; 4 Laboratory of Human Genetics and Medicine, Federal University of Pará, Belém, Pará, Brazil; University of Texas Health Science Center at Houston, United States of America

## Abstract

*MYH9* polymorphisms have been described to be associated with the risk of CKD in non-diabetic nephropathy, HIV nephropathy and FSGS. Predominating in black descendants, *MHY9* genetic variants could partially explain the excess risk of CKD associated with African ancestry. However, recent data suggests that *APOL1* gene co-segregate with *MYH9*, and could be the gene truly associated with CKD risk. In this study, we evaluated the role of *MYH9* and *APOL1* gene polymorphisms in the risk of CKD in Brazilian patients with lupus nephritis (LN). A retrospective analysis of 196 LN patients was done. *MYH9* rs4821480, rs2032487, rs4821481 and rs3752462, *APOL 1*rs73885319, rs16996616, rs60910145, rs71785313, and *APOL3* rs11089781 gene polymorphisms were determined. Genetic ancestry was ascertained both by autossomal ancestry and mitochondrial haplogroup. Primary outcome was defined as doubling of serum creatinine (DC) or end stage renal disease (ESRD). Sixty-two patients presented the PO. In our population, *MYH9* and *APOL1* were not in LD. None *APOL* polymorphism was associated with the PO, whereas rs3752462 *MYH9* polymorphism showed a positive association (HR3.72, 95%CI 1.47–9.38, p = 0.005). When we analyzed the *MYH9* E1 haplotype, the GCCT carriers (1 or 2 alelles present in 29.7% in the PO group vs. 18.5% in controls) showed a significant association to the risk of PO, even after adjustments for baseline estimated creatinine clearance and autossomal ancestry (HR 2.0, 95%CI 1.2–3.4, p = 0.01). Our results show that in our population *MYH9*, but not *APOL1*, gene polymorphisms confer an increased risk of CKD in LN patients, independently of race.

## Introduction

Polymorphisms in the non-muscle heavy chain 9 (*MYH9*) gene have been associated to the risk of chronic kidney disease (CKD) in populations with non-diabetic kidney disease, HIVAN, and idiopathic FSGS [1]–[4]. The risk variants are particularly common in African descendants and *MYH9* polymorphisms have been proposed to contribute to approximately 70% of non-diabetic forms of ESRD in African Americans (AA) and to 40 to 45% of all ESRD in this ethnic group [5]. These first results led to the hypothesis that MYH9 could be a major determinant of the excess risk of CKD associated with African ancestry.

However, in 2010, it was proposed that *APOL1* gene polymorphisms were more intensely associated to the CKD risk, previously attributed to *MYH9* [6], [7]. The two genes co-segregate in many populations making it difficult to differentiate between the two association signals. In addition, *MYH9* gene polymorphism was not shown to be associated with CKD in non-African populations, such as in American Indians [8].

African ancestry has also been proposed to be associated with a higher incidence of CKD in lupus nephritis (LN) [9]. However, *MYH9* polymorphisms have not been shown to be associated with ESRD in African Americans patients with LN [10]. Recently, *MYH9* polymorphisms (but not *APOL1*) have been implicated in the risk of LN in European Americans and in Gullah populations. No association was found between LN and *MYH9* in AAs (confirming the previous study), Asians, Amerindians or Hispanics [11].

The objective of our study was to evaluate the relationship between *MYH9* and *APOL1* gene polymorphisms and the risk of CKD in LN Brazilian patients, a highly admixtured population.

## Study Population and Methods

### Patients and protocol

Initially, all adult patients with a renal-biopsy confirmed diagnosis of LN undergoing regular follow-up in the Nephrology Division between July 2005 and July 2007 were enrolled. Patients with diabetes, hepatitis B, hepatitis C, HIV or those who had less than 3 months of follow-up were excluded, leaving for the analysis 196 female patients.

All patients fulfilled the American College of Rheumatology 1982 revised criteria [12]. LN was determined according to the WHO classification [13]. Treatment was determined by the attending physician. The most common therapeutic regimen for patients with class IV disease was intravenous and/or oral corticosteroids associated with intravenous cyclophosphamide (more than 90% of class IV nephritis), administered according to the US National Institutes of Health protocol [14]. For those with a good response at 6 months of treatment, conversion to azathioprine or mycophenolate mofetil was common. Patients with class V LN were mostly treated with oral corticosteroids and/or cytotoxic drugs [15] (approximately 50% of patients received cyclophosphamide and corticosteroids). Laboratory testing was periodically performed to determine serum creatinine, complement, antinuclear antibody (ANA), anti-double stranded DNA (dsDNA), blood cell counts, proteinuria, and urinalysis. ANA was determined by immunofluorescence in Hep-2 cells, anti-dsDNA by immunofluorescence using *Crithidia luciliae* as substrate, and complement by radial immunodiffusion.

Race was assessed by three different ways: a physician-assessed classification of skin color, based solely on a visual and subjective estimation of the ancestry of the patient, as done before [16]; by mitochondrial haplogroup and by autosomal genetic ancestry [17].

Disease activity was assessed using the SLE Disease Activity Index (SLEDAI) and renal disease activity by the renal SLEDAI score [18]. Renal flare was defined as (i) the recurrence or the development of nephrotic syndrome (serum albumin ≤3.5 g/dl and 24 h proteinuria ≥3 g; (ii) renal impairment (≥33% increase of serum creatinine within a 1-month period directly attributed to lupus and confirmed 1 week later; flare referred to as ‘nephritic flare’) or (iii) a threefold increase of 24 h proteinuria within a 3-month period accompanied by microscopic haematuria (defined as a number of red blood cells (RBC) per high power field superior to upper normal limit for the local laboratory) and ≥33% reduction of serum C3 level within a 3-month period, only to those patients with low grade baseline 24 h proteinuria (≥0.5 g and <1 g). Renal remission was defined as a serum creatinine ≤1.4 mg/dl and a 24 h proteinuria <0.3 g and a urinary RBC count <10/high power field, at any time of follow-up. Partial remission was defined by a 24 h proteinuria between 0.3 and 2.9 g, or a proteinuria drop >50% from the baseline, at any time of follow-up. The renal function should be stable [19].

Primary outcome (PO) was defined as duplication of serum creatinine or need of renal replacement therapy. PO status was defined by data collected at baseline (around one week before renal biopsy) and at the last follow-up examination until to December 2010.

The study was approved by the Research Ethics Committee of our hospital (Comissão de Ética para Análise de Projetos de Pesquisa – CAPPesq – Hospital das Clínicas – Faculdade de Medicina da Universidade de São Paulo) and all patients provided written informed consent.

### SNP Selection and Genotyping

Genomic DNA from subjects was extracted from peripheral blood following standard salting-out procedure [20]. Polymorphisms for the MYH9 rs4821480, rs2032487, rs4821481, rs3752462 (E1 haplotype), for APOL1 rs73885319, rs16996616, rs60910145, rs71785313, and APOL3 rs11089781 were determined by high resolution melting technique (HRM) (Rotor Gene 6000, Qiagen, Courtaboeuf, France). The QIAgility (Qiagen, Courtaboeuf, France) is an automated instrument that was used to optimize the sample preparation step in the PCR-HRM. In each genotyping assay, 96 samples were genotyped in a specific disc, and samples of three pattern genotypes inserted. Polymorphic regions were amplified by adding 1 µl of genomic DNA to asolution of 10 µMTris-HCl (pH = 9), 50 mMKCl, 2.5 mM MgCl2, 100 mM of each dNTP, 0.3 U of Easy Taq DNA Polymerase (Invitrogen), and 5 pmol of each primer. Amplification of the fragments was performed using the primer sense

rs4821481F: CTCACGGCTGGCAAAGAAGAGCTTTC;

rs4821481R: AGAGGGGAAAGGACAAACCCTTCCC;

rs2032487F: AGAGGCTGCCACACGGCGCTCACCTG;

rs2032487R: GCCACCAGGCCACCTTCTCCGTGCC;

rs4821480F: ATTTTCCTAGATCAAAGGATAATTTT;

rs4821480R: AAAGGTCACGAGCTCCCCTGAAACA;

rs3752462F: CAGGTGTGAGGTCAAAGCAAGCCTGG;

rs3752462R: ACTCACTGGCTTCTCAATGAGGTCG;

rs73885319F: CCTGGAAATGAGCAGAGGAG;

rs73885319R: CATCCAGCACAAGAAAGAAGC;

rs60910145F: CTCAGGAGCTGGAGGAGAAG;

rs60910145R: GCCCTGTGGTCACAGTTCTT;

rs11089781F: TGGAGAACGTGTCTGGTTATTA;

rs11089781R: GCAAGATCCAGCTGTTCTGA;

A 30-cycle PCR was carried out using the following conditions: denaturation of the template DNA for first cycle at 94°C for 120 s, denaturation at 94°C for 20 s, specific annealing temperature for 20 s, and extension at 72°C for 22 s. PCR was performed using a 10 µL reactive solution (10 mMTris–HCl, 50 mMKCl, pH 9.0; 2.0 mM MgCl2; 200 µM of each dNTP; 0.5 U Taq DNA Polymerase; 200 nM of each primer; 10 ng of genomic DNA template) with addition of fluorescent DNA intercalating SYTO9 (1.5 µM; Invitrogen, Carlsbad, USA).

In the HRM phase, the Rotor Gene 6000 measured the fluorescence in each 0.1°C temperature increase in the range of 70–90°C. Melting curve was generated by the decrease in fluorescence with the increase in the temperature; and in analysis, nucleotide changes results in three different curve patterns. Samples of the three observed curves were sequenced (ABI Terminator Sequencing Kit and ABI 3500XL Sequencer—Applied Biosystems, Foster City, CA, USA) to confirm the genotypes indicated by HRM analysis. In addition, 10% of the samples were reanalyzed as quality control and gave identical results.

### Statistical analysis

Mann-Whitney and chi-square tests were used for comparison among continuous and categorical variables, respectively. Hardy-Weinberg equilibrium for the genotypes was tested by chi-square goodness-of-fittest. Linkage Disequillibrium (LD) was estimated using r2 and χ2 statistics using Haploview 3.32. Hapstat (http://www.bios.unc.edu/~dlin/hapstat/) was used for comparisons of SNPs and frequency of the PO, using dominant, additive, and recessive models. Cox proportional hazard models (SPSS v13.0) were built for the risk of the PO according to *MYH9* and *APOL1* genotypes and also according to the *MYH9* E1 haplotype (determined using Haploview). Kaplan-Meier curve of the PO according to MYH9 E1 haplotype was created and log-rank test calculated. All tests were two-sided and considered significant if p value<0.05.

## Results

In Table 1, baseline clinical, laboratorial and histological data of the 196 female patients with LN are shown. Patients were young, and presented high creatinine, low estimated creatinine clearance, high proteinuria and 66% of proliferative forms of LN. Of the 196 patients enrolled, 62 presented the PO (end-stage renal disease or creatinine duplication), with a mean follow-up time of 6.2 years. As expected, patients who presented the PO had a higher percentage of hypertension, lower C3 level, high serum creatinine and lower estimated creatinine clearance and higher percentage of proliferative forms than those without the PO. In addition, chronicity index, number of fibroblastic crescents and tubule-interstitial fibrosis at baseline were significantly higher in those who presented the PO. Poor response to treatment and occurrence of renal flares were also more common in the PO group. Interestingly, only one third of the population self-reported race as black or mulatto (nearly 65% self-reported race as white), with no difference according to the PO. Analyzing data of mitochondrial haplogroup, there was approximately 50% of African, 30% of Ameridian and less than 15% European ancestry in our sample. This distribution was not different according to PO (p = 0.42). Autossomal ancestry was not different among groups either, with approximately 30% of African genes present in our sample.

**Table 1:** Baseline clinical, laboratorial and histological data of 196 LN patients and according to renal outcome.

	All patients (n = 196)		non ESRD-CD (n = 134)		ESRD-CD (n = 62)		p*
**Age (years; mean/std)**	28.6	9.8	28.5	9.5	28.9	10.5	0.88
**Physician-assessed skin color (black+mulatto; n/%)**	64	32.6	45.0	33.6	19	30.6	0.68
**Hypertension (yes; n/%)**	151	77.0	95	70.9	56	90.3	0.003
**Creatinine (mg/dL; mean/std)**	1.6	0.95–3.1	2.0	1.6	3.8	3.1	<0.0001
**Est. creatinine clearance (MDRD, ml/min/1.73 m^2^; median/IQR)**	50.8	39.7	48.3	26.1–85.0	23.2	9.0–58.7	<0.0001
**Albumin (mg/dL; mean/std)**	2.6	0.8	2.6	0.9	2.6	0.8	0.95
**24 h proteinuria (g/d; median/IQR)**	3.9	2.2–6.8	3.9	2.3–6.5	4.4	1.7–7.0	0.65
**ANA (positive >1/80; n/%)**	161	0.82	110	82.1	51	82.3	0.98
**C3 (mg/dL; median/IQR)**	58	41.5–81.5	63	42.3–86	51	39.5–67.5	0.03
**C4 (mg/dL; median/IQR)**	11	6.2–20	12	6.6–20	11	6–18	0.77
**SLEDAI (mean/std)**	21.0	6.4	20.4	5.9	22.2	7.2	0.25
**RENAL SLEDAI (mean/std)**	9.1	3.4	8.9	3.6	9.7	2.8	0.08
**Lupus nephritis histologic classification (n/%)**							
***III+IV***	130	66.3	84	62.7	46	74.2	0.01
***V***	61	31.1	49	36.6	12	19.4	
***VI***	5	2.6	1	0.7	4	6.5	
**Activity (median/IQR)**	4	3–5	4	3–6	4	3–5	0.93
**Chronicity (median/IQR)**	3	0–5	2	0–4	5	2–7	<0.0001
**Number of epithelial crescents (median/IQR)**	0	0–1	0	0–1	0	0–2	0.35
**Number of fibroblastic crescents (median/IQR)**	0	0–1	0	0–1	0	0–2	0.01
**Tubulo-interstitial fibrosis (>50% fibrosis; n/%)**	18	9.5	6	4.5	12	21.8	0.001
**Vessels (presence of abnormalities; n/%)**	69	35.2	41	30.6	28	45.2	0.05
**Renal flare (nephritic or nephrotic; n/%)**	93	47.4	63	53.8	30	81.1	0.003
**Response to treatment** ** **(n/%)**							
***complete remission***	136	72.7	118	88.1	18	34.0	<0.0001
***partial remission***	25	13.4	13	9.7	12	22.6	
***no response***	26	13.9	3	2.2	23	43.4	
**Race by mitochondrial haplogroup (n/%)**							
***African***	91	46.4	60	46.5	31	56.4	0.42
***Ameridian***	62	31.6	45	34.9	17	30.9	
***European***	31	15.8	24	18.6	7	12.7	
**Race by autossomal ancestry (mean/std)**							
***African***	0.31	0.17	0.29	0.17	0.33	0.18	0.23
***Ameridian***	0.16	0.09	0.16	0.09	0.17	0.08	0.11
***European***	0.53	0.18	0.55	0.18	0.49	0.17	0.11

* p = Mann-Whitney or chi-square.

** 9 patients were excluded because treatment was not performed or interrupted.

IQR, interquartile range.

**Figure 1:**
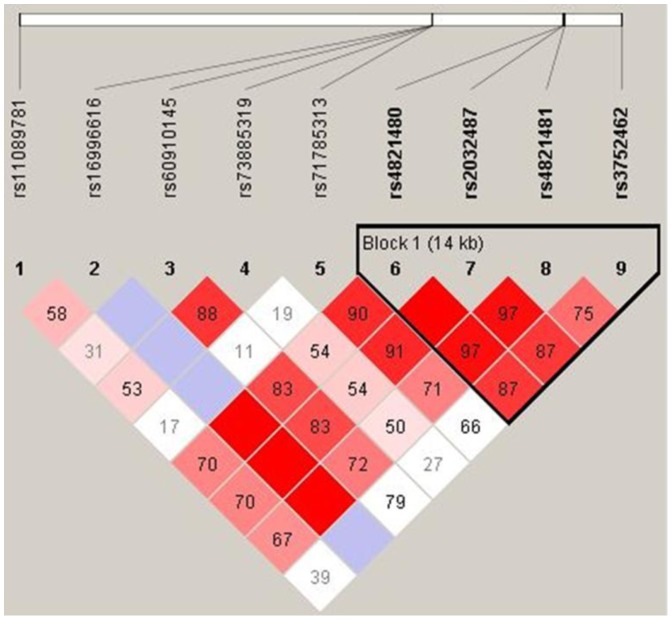
Linkage disequilibrium structure of *MYH9* and *APOL1* gene polymorphisms in 196 LN Brazilian patients.

In our sample, linkage disequilibrium structure of the *MYH9* and *APOL 1* (Figure 1) showed that the four *MYH9* polymorphisms segregate as an haplotype. Importantly, *APOL* markers were not in linkage disequilibrium with *MYH9* LD block.

Firstly, we analysed the association between each polymorphism and the risk of PO (Table 2). In these analyses, only rs3752462 polymorphism was associated with the PO (using a recessive model), with an adjusted HR of 3.72 (1.47–9.38, p = 0.005) for the risk of CKD (Table 3).

**Table 2:** Risk of the PO according to MYH9 and APOL gene polymorphisms in 196 LN patients.

	non ESRD-CD (n = 134)		ESRD-CD (n = 62)		p*
**Genotypes MYH9**					
**rs4821480**					
***GG***	75	56.0	33	54.1	0.93
***GT***	47	35.1	23	37.7	
***TT***	12	9.0	5	8.2	
**rs2032487**					
***CC***	75	56.4	33	54.1	0.91
***CT***	46	34.6	23	37.7	
***TT***	12	9.0	5	8.2	
**rs4821481**					
***CC***	71	53.4	30	49.2	0.75
***CT***	47	35.3	25	41.0	
***TT***	15	11.3	6	9.8	
**rs3752462**					
***CC***	44	33.1	7	11.7	0.01
***TC***	61	45.9	36	60.0	
***TT***	28	21.1	17	28.3	
**Genotypes ApoL 1 and 3**					
**rs73885319**					
***AA***	110	85.3	51	86.4	0.33
***AG***	18	14.0	6	10.2	
***GG***	1	0.8	2	3.4	
**rs16996616**					0.69
***GA***	6	4.7	2	3.4	
***GG***	123	95.3	57	96.6	
					0.77
***TG***	15	11.5	6	10.0	
***TT***	116	88.5	54	90.0	
**rs71785313**					
***DD***	2	1.5	1	1.7	0.36
***ID***	6	4.6	6	10.0	
***II***	123	93.9	53	88.3	
**rs11089781**					
***AA***	1	0.8	1	1.7	0.83
***GA***	13	9.8	6	10.3	
***GG***	118	89.4	51	87.9	

* p = chi-square.

**Table 3:** Unadjusted and adjusted Cox proportional hazard models on the risk of CKD according to rs3752462 MYH9 polymorphism.

rs3752462	HR	95% HR		p*
**TC+TT vs. CC**	2.89	1.31	6.36	0.008
*Adjusted for MDRD, hypertension and african autossomal ancestry*				
**TC+TT vs. CC**	3.72	1.47	9.38	0.005

* Cox proportional hazard models.

**Figure 2:**
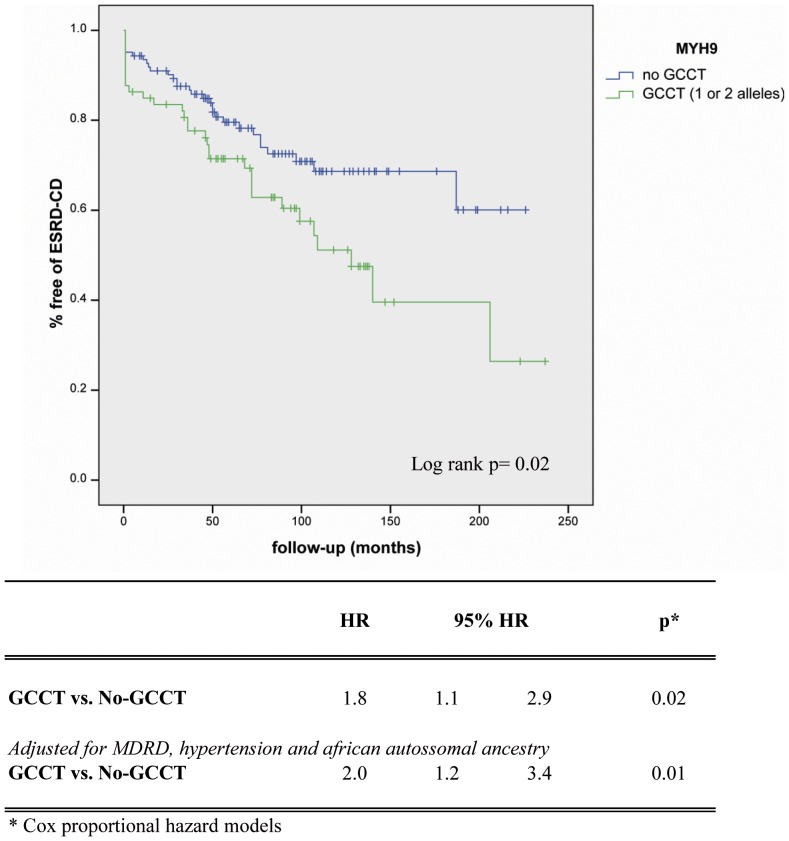
Cox proportional hazard models and Kaplan-Meier curve of the risk of the PO according to *MYH9* E1 haplotype.

*MYH9* E1 haplotype (homo or heterozygous) was present in 29.7% of cases and in 18.5% of controls. By Cox regression analyses, presence of at least one *MYH9* E1 haplotype was significantly associated with the risk of the PO (HR 1.8, 95% CI 1.1–2.9, p = 0.02; Figure 2). After adjustment for estimated creatinine clearance and African autossomal ancestry, *MYH9* E1 haplotype remained associated to the risk of CKD (HR 2.0, 95% CI 1.2–3.4, p = 0.01). Kaplan Meier curve is also shown in Figure 2, with a log rank p value of 0.02. No difference in the model was shown by repeating the analyses with adjustment for mitochondrial haplogroup and also by including both African and Ameridian or European ancestry as covariates.

Lastly, we analysed clinical and laboratorial baseline data according to *MYH9* E1 haplotype, in order to evaluate if genotype was related to lupus activity (Table 4). The only difference found was that people with the GCCT haplotype presented a higher percentage of class VI LN, suggesting that the haplotype is not related to lupus activity, but only to the risk of chronicity and CKD. Interestingly, the degree of proteinuria was not correlated with the GCCT group.

**Table 4:** Baseline clinical, laboratorial and histological data according to *MYH9* E1 haplotype groups.

	non GCCT (n = 123)		GCCT (n = 73)		p*
**Age (years; mean/std)**	27.8	9.3	30	10.5	0.15
**Physician-assessed skin color (black+mulatto; n/%)**	43	35.0	21	28.8	0.37
**Hypertension (yes; n/%)**	93	75.6	58	79.5	0.54
**Creatinine (mg/dL; mean/std)**	2.5	2.3	2.6	2.4	0.79
**Est. creatinine clearance (MDRD, ml/min/1.73 m^2^; median/IQR)**	39.5	19.3–83.6	41.3	17.9	0.70
**Albumin (mg/dL; mean/std)**	2.6	0.9	2.6	0.8	0.68
**24 h proteinuria (g/d; median/IQR)**	3.9	2.3–6.9	3.9	1.9–6.4	0.75
**ANA (positive >1/80; n/%)**	104	84.6	57	78.1	0.25
**C3 (mg/dL; median/IQR)**	62.0	42–86	52.8	40–78	0.14
**C4 (mg/dL; median/IQR)**	11.0	6–20	12.0	7–18	0.76
**SLEDAI (mean/std)**	20.7	6.6	21.4	6.1	0.60
**RENAL SLEDAI (mean/std)**	9.1	3.5	9.2	3.2	0.74
**Lupus nephritis histologic classification (n/%)**					0.01
***III+IV***	82	66.7	48	65.8	
***V***	41	33.3	20	27.4	
***VI***	0	0.0	5	6.8	
**Activity (median/IQR)**	4	3–5	4	3–6	0.66
**Chronicity (median/IQR)**	2	1–5	3	0–6	0.29
**Number of epithelial crescents (median/IQR)**	0	0–1	0	0–2	0.83
**Number of fibroblastic crescents (median/IQR)**	0	0–1	0	0–2	0.47
**Tubulo-interstitial fibrosis (>50% fibrosis; n/%)**	8	6.8	10	13.9	0.11
**Vessels (presence of abnormalities; n/%)**	48.0	39.0	21.0	28.8	0.15
**Renal flare (nephritic or nephrotic; n/%)**	58	58.6	35	63.6	0.54
**Response to treatment** ** **(n/%)**					0.22
***complete remission***	89	74.2	47	70.1	
***partial remission***	18	15.0	7	10.4	
***no response***	13	10.8	13	19.4	
**Race by mitochondrial haplogroup (n/%)**					0.94
***African***	56	50.0	35	48.6	
***Ameridian***	38	33.9	24	33.3	
***European***	18	16.1	13	18.1	
**Race by autossomal ancestry (mean/std)**					
***African***	0.32	0.17	0.28	0.17	0.08
***Ameridian***	0.16	0.09	0.17	0.09	0.54
***European***	0.52	0.18	0.55	0.17	0.22

* p = Mann-Whitney or chi-square.

** 9 patients were excluded because treatment was not performed or interrupted.

IQR, interquartile range.

## Discussion

The first association between *MYH9* and kidney disease derived from the observation that patients with the giant platelet syndromes, a group of diseases related to *MYH9* mutations and with a spectrum of abnormalities including low platelet count, giant platelets, hearing loss, and cataract, may present FSGS. In 2008, a genome-wide association study proposed that *MYH9* was very strongly associated to HIVAN and idiopathic FSGS [4]. This finding was later confirmed in non-diabetic nephropathy in African Americans [1] and even in the setting of diabetic nephropathy [21]. This led to the hypothesis that *MYH9* could be the gene majorly determinant of the excess risk of CKD in African descendants. However, in 2010, two studies [6], [7] re-evaluated this initial finding and suggested that *APOL1*, a neighbor gene presenting very strong co-segregation with *MYH9* in African descendants, presented an even stronger association to CKD than *MYH9*, being the marker possibly responsible for the effect previously attributed to *MYH9* [22]. It was proposed that the *APOL1* variants (called G1 haplotype, related to rs73885319 and rs60910145 polymorphisms, the most frequent type and G2, related to rs71785313 mutation), mutually exclusive (too close for recombination), suffered natural selection in Africa due to an adaptative response related to trypanossomiasis, particularly to some resistant forms prevalent in the sub-Saharan Africa *(Trypanosomo brucei rhodesiense* and *gambiense*). *APOL1* is known to be a trypanolytic factor. Resistant forms of trypanossomisis exhibit a protein (SRA) that interacts with *APOL1*, reducing its trypanolytic activity. Serum of patients carrying the G1 and G2 variants presented a higher lytic ability than serum of those patients not carrying these variants [7]. Another interesting fact is that the *APOL1* variants were not found in HIV Ethiopian individuals, described previously for the absence of HIVAN [23]. APOL1 has now been demonstrated to be related to HIVAN and FSGS [24], to collapsing nephropathy in lupus patients [25] and to the younger age of initiation of dialysis [26].

In LN, data is scarce and controversial. In the African American population, despite the high frequency of *MYH9*, no association was found [10]. This first study did not test *APOL1* genes. A more recent study analysed the role of *APOL1* and *MYH9* polymorphisms in African and non-African SLE patients [11]. When compared to healthy controls, *MYH9* was related to LN only in European Americans and in Gullah descendants. No association was found for African American, Hispanics, Asian and Amerindians, even in a sub-analysis including those patients with LN and ESRD. In addition, in this study, the *APOL1* polymorphisms were not associated with LN.

Our results suggest that *MYH9* polymorphisms, and not *APOL1*, are associated with an increased risk of CKD in patients with LN. Interestingly, in this Brazilian sample, the two genes were not in LD. The frequency of the risk alleles of both *MYH9* (29.7% vs. 18.5% in case and controls, respectively) and *APOL1* (approximately 10%) was not as high as those seen in the African American population. Although we could detect a positive association of *MYH9* with ESRD, it is possible that the absence of association between the PO and *APOL1* genes in our study could be due to a relatively small sample size and lack of power, leading to a type II error. However, if the association were solely due to alleles located in the APOL1 gene and not to alleles located in the MYH9 gene, we should have found no association in MYH9 region, that, as we have shown, is independent of the APOL1 region in our sample. Our interpretation of the results is that there is indeed an association signal residing within the MYH9 region. This, by no means imply a causal role of the protein encoded by MYH9 over the studied phenotype. There are several possibilities, including the existence of genetic variants in this genomic region that affect distant genes, even APOL1. In addition, we were able to adjust our analysis by three different, but correlated, measures of ethnicity: physician-assessed skin color, mitochondrial ancestry and autosomal genetic ancestry.

Another limitation of our study is that it was not an inception cohort and this may lead to prognosis bias, since severe cases are more likely to be included, particularly in a tertiary hospital such as ours. However, this bias is likely to be equally distributed among genotypes. Moreover, race, an important determinant of social-educational status and access to medical care, was not statistically different among genotypes groups.

Lastly, since several SNPs were tested, our results can be criticized considering we performed multiple comparisons, a strategy which may increase the odds of a positive association by random. A correction for this effect would be the best approach, but these corrections are too conservative for the sample size we had in this sample.

In our study, the E1 haplotype was not related to any SLE clinical or laboratorial marker of activity. This fact suggests that the MYH9-APOL1 gene region is probably acting in the risk of CKD not through immunological mechanisms and activation of nephritis itself, but probably through the disregulation of podocyte function (the main cell type so far considered to be related to MYH9-APOL1 genes), with enhancement of proteinuria and glomerulosclerosis.

Several questions arise from all these initial studies regarding the MYH9-APOL1 gene region. 1. The polymorphisms described both for APOL1 genes and MYH9 occur in non-coding areas, and it is still not well understood how these mutations are related to phenotypic changes. One strong possibility is that the mutations modulate protein transcription. This process could either be on the protein itself (a genetic variant in the MYH9 regulating the expression of the MYH9 protein), or on a more distant protein (such as APOL1). This hypothesis, however, needs further confirmation with animal and clinical studies evaluating gene expression. 2. The data gathered so far points to APOL1 as the gene truly involved in the risk of CKD [22]. However, it is necessary to understand how APOL1 could be related to CKD pathogenesis. Scarce data on biological mechanisms are available and evidence still favors MYH9. MYH9 codifies the myosin-IIa, an enzyme present in the podocyte foot process and known to be related to filament movement. In animal studies, mutations in MYH9 are related to phenotypic kidney abnormalities including albuminuria and FSGS [27], [28], as well as defects in morphogenesis [29]. HIV-1 downregulates the expression of MYH9 in transgenic mice, a finding confirmed in humans [30]. In addition, MYH9 mutation is related to FSGS in the giant platelet syndromes, a clinical fact that suggests that this protein actually has a role in the podocyte biology.

On the other hand, insights on APOL1 are available from recent data. APOL1 is more strongly expressed in the placent, lung and liver [31], as well as in heart, pancreas and endothelial cells. Recently, it was shown that APOL1 is also expressed in the glomeruli, with a signal localizing at the cytoplasm of podocytes [32]. It is known to regulate cell death pathways [33], a property possibly related to its trypanolitic activity. A recent study in autopsies showed that APOL1 risk allele carriers presented glomerular and kidney hypertrophy and accelerated nephron loss when compared to controls [34]. In HIVAN, APOL1 G1 and G2 polymorphism has been related to proteinuria [35].

It is clear that MYH9-APOL1-related kidney disease is a very exciting new area in Nephrology. First, it could provide the first genetic tool allowing the identification of patients at increased risk for ESRD in a complex polygenic disease such as CKD, at least in populations in whom the mutations are known to be prevalent. Secondly, comprehension of the biological mechanisms determining proteinuria and CKD in patients presenting these mutations can create opportunity for new therapeutic targets and measures.

In conclusion, our study suggests that MYH9 gene polymorphisms and the E1 haplotype are related to the risk of CKD in LN in an admixture population. APOL1 was not related to this risk in our sample, but this result can be related to our small sample size. Comprehension of the biological mechanisms underlying this association is a current challenge for future research.
